# Refractory Urinary Incontinence in Girls: The Role of the Bladder Neck

**DOI:** 10.3389/fped.2017.00074

**Published:** 2017-04-10

**Authors:** Rafal Chrzan

**Affiliations:** ^1^Pediatric Urology, Jagiellonian University Medical College, Krakow, Poland

**Keywords:** lower urinary tract symptoms, urinary incontinence, bladder neck, children, etiology

## Abstract

**Background:**

Prevalence of lower urinary tract dysfunction (LUTD) in children is between 6 and 9% with urinary incontinence (UI) being one of the most common symptom.

**Various aspects of lower urinary tract symptoms (LUTS):**

Anatomical anomalies of the urinary tract as well as neurogenic underlying pathology can results in LUTS. Comorbidities and long-term consequences of the LUTD for the female patients as well as genetic issues are also briefly discussed.

**The role of the bladder neck:**

Thanks to urodynamics, we have learnt a lot about the lower urinary tract function, but the role of the bladder neck in the pathophysiology of LUTS in children is not clear. Secondary bladder neck hypertrophy is a well-described pathology, but there is no standardized treatment for this phenomenon. Primary bladder neck dysfunction has already been defined by the International Children’s Continence Society.

**Refractory UI in girls:**

Uniform diagnostic protocols are used in these girls with UI. Treatment consists of standard urotherapy, additional interventions, and pharmacotherapy in selected cases. Those with refractory UI require careful reassessment to look for the unrecognized disorders. Invasive urodynamics should be done in those patients. Ultrasound of the bladder neck region and the pelvic floor can be helpful, but its interpretation is very subjective. In a small group bladder neck insufficiency can be found and those might benefit from a surgical intervention.

**Future perspective:**

Strict criteria of the bladder neck insufficiency in children must be defined. Early surgical intervention in girls with bladder neck insufficiency might reduce the long period of intensive conservative treatment.

## Introduction

The prevalence of lower urinary tract symptoms (LUTS) in school-age children is approximately 6–9% (1–3). They usually seek help because of recurrent urinary tract infections (UTIs) and/or urinary incontinence (UI). Subtle symptoms like voiding postponement, hesitancy, or bladder overactivity are usually neglected for a long period by the child and his/her environment. It is said that “the child will grow out of the problem.”

Uniform protocols have been made to diagnose and treat LUTS in children (4–6). Although the results of conservative treatment are satisfactory, a small number of patients suffer from refractory incontinence (7–9). Our primary results indicate that bladder neck insufficiency can lead to persistent LUTS in girls, and surgical treatment can be effective in this group (10).

This perspective paper is to discuss the etiology of lower urinary tract dysfunction (LUTD) and the role of the bladder neck in the female patients with refractory UI.

## Various Aspect of LUTD

### Anatomical Anomalies and Neurogenic LUTD

During the last three decades, we have learnt a lot about the physiology of micturition and maturation of the lower urinary tract function (3). Unaffected anatomy of the bladder and its outlet is essential to become continent. Exstrophy–epispadias complex, bilateral ureteral ectopy, and several other congenital anomalies result in continuous UI and require surgical treatment (11). Some of those patients might need additional urotherapy. Proper innervation of the lower urinary tract is crucial for its normal function and good coordination between the bladder and the sphincter mechanism (3). Patients with neurogenic LUTD need close follow-up in a multidisciplinary setting to reduce the risk of damage of the upper urinary tract (12, 13). Clean intermittent catheterization and antimuscarinics are the first-line therapy and are highly effective in the most of the cases. Provided the renal function is preserved, surgical intervention targeting the bladder and/or the bladder outlet can be done to improve dryness on the long term (14–16).

Careful reevaluation of all patients with refractory UI should take place to exclude overlooked anatomical anomalies and/or underlying neurogenic pathology.

### Consequences of LUTD

The pathophysiology of LUTS in children is still not fully understood. Bowel dysfunction can be found in 1/3 of patients with LUTS (17). Coexistence of the recurrent UTIs, LUTS, and vesicourethral reflux is a well-known clinical finding and can result in chronic renal failure (18, 19). Young women are prone to this worst-case scenario as impairment of the renal function can occur during pregnancy. A thorough approach is needed to treat those patients in the proper way early in childhood. Over the years, uniform therapeutic protocols have been developed in children with LUTS consisting of antibiotic prophylaxis for infections, antimuscarinics for overactive bladders, cognitive and biofeedback urotherapy for dysfunctional voiding, physiotherapy in case of insufficient pelvic floor control, and the treatment of coexisting constipation (7, 17, 20).

### LUTS in Female Patients and Genetics

Family studies and twin studies demonstrate that LUTS and pelvic organ prolapse in females are heritable. Having a first-degree relative with incontinence or prolapse is associated with approximately a threefold increased risk for the development either condition (21–23). Stress UI is usually associated with a progressive weakening of the pelvic floor, and multigravidas were thought to be at higher risk. On the other hand, not all women after vaginal deliveries are incontinent and not only those who have given birth suffer from UI (24). It seems that pelvic floor and bladder neck insufficiency can be congenital, and a genetic factor can play an important role in the development of LUTS. Identification of the genetic variants underlying the heritability of these conditions might provide useful markers for estimation of the clinical risk of UI in the future.

## Bladder Neck and LUTS

### Secondary Bladder Neck Hypertrophy

Bladder outlet obstruction is a well-described phenomenon in men and is usually related to benign prostate hypertrophy. Pharmacological treatment can be offered to those patients to reduce the voiding as well as the storage symptoms (25). Historically alpha_1_-blockers were mainly used to relieve the voiding symptoms, but the latest studies showed that non-selective antimuscarinics can reduce the storage symptoms acting through the M_3_ receptor and voiding symptoms through the M_1–2_ blockade (26–28).

Secondary bladder neck hypertrophy can be found in children with infravesical obstruction. There is a general consensus that in boys with posterior urethral valves bladder neck must be evaluated and treated to enable optimal bladder emptying on the long term (29). Alpha_1_-blockers, bladder neck incision, and botulinum toxin type A injection into the bladder neck have been reported as treatment options for this condition, but no randomized trials are available (30–32).

### Primary Bladder Neck Dysfunction

The terminology of physiological micturition and detailed descriptions of LUTS in children have been proposed by the International Children’s Continence Society (33). In the latest version of the standardization document, primary bladder neck dysfunction has been mentioned for the first time. This entity is characterized by a delayed opening of the bladder neck at the beginning of the voiding phase, which is called a prolonged *lag time*. The *lag time* can be measured during invasive urodynamics and/or by means of uroflowmetry combined with electromyography (EMG) of the pelvic floor. However, the literature on this topic is sparse (34). Alpha-blockers have been used in those patients, but only few trials were done to investigate this issue (35–37). Nevertheless, in the majority of the patients an overactive pelvic floor is the primary cause of the functional infravesical obstruction leading to LUTS.

## Functional Bladder Neck Insufficiency in Girls: An Unrecognized Condition?

Urinary incontinence is a symptom related to the storage phase. Intermittent UI in girls is predominantly a functional problem as a result of abnormal detrusor or/and sphincter function. Standardized tools are used to make the diagnosis including bowel and bladder diary, validated questionnaires, thorough physical examination, urinalysis, and uroflowmetry with post void residual assessment. Urotherapy should be introduced first in patients with UI followed by pharmacological treatment in selective cases. Unfortunately, not all of them can be freed of symptoms. Stress incontinence or stress-induced urge incontinence is a very rare condition in childhood. Girls who present with refractory UI need special attention and an extended checkup including the bladder neck function that must be done. Generalized hyperlaxicity of joints should be looked for in children who do not respond to the initial treatment; however, the evidence of its importance is rather weak.

The diagnostic process should be as minimally invasive as possible. This is of a special importance in children. EMG in combination with uroflowmetry can be helpful to measure pelvic floor activity and to estimate the *lag time* (34). Ultrasonography is widely used to assess the urinary tract. Dynamic ultrasound of the pelvic floor in the pediatric population has been described, but this tool is used only in a few centers (38, 39). (Video)-urodynamics is done in patients with refractory UI for better assessment of the anatomy and the function of the bladder, the bladder neck, and the pelvic floor. However, VUDS has its limitations because UI reported by the patient is not always reproducible during this study (40).

Functional bladder neck insufficiency in children (FuBNiC) has not been defined to date. Continuous open bladder neck during the filling phase and its hypermobility during straining/voding can result in LUTS. Overactive bladder is usually associated with a small volume in relation to age (8, 33). In children with FuBNiC, urge symptoms are present, but the bladder capacity is adequate to the normal range for age. This hypothesis needs further prospective evaluation. Uroflowmetry in those patients can probably be characterized by a short flow time and a high maximal flow rate (a so-called “tower-shaped curve”). The short *lag time* can appear as a result of a sudden opening of the pelvic floor during strong detrusor contraction provoked by the open bladder neck. Some authors claim that the tower-shaped voiding curve is pathognomonic for overactive bladder, which can obviously be true, but it does not explain the underlying pathology (41). The preliminary data showed that the bladder neck insufficiency can be evaluated by means of a perineal ultrasound and a video-urodynamic study. Both investigations can detect an open bladder neck during filling and a cystocele (mobile bladder neck) during straining (10). Ultrasound is preferable in children because it is less invasive.

The question arises whether bladder neck insufficiency in children is an anatomical or a functional condition. Regardless the exact pathophysiology, a small series showed that 50–75% of female patients with refractory UI and insufficient bladder neck can be cured by a colposuspension procedure (10, 42). The most important question is how to select the patients for the surgical treatment.

Based on the personal experience and the preliminary reports from the literature, diagnostic criteria for FuBNiC might be as follows:

- Clinical:
  1 UI
  2 Symptoms of bladder overactivity in patients with adequate bladder volume for age
  3 Recurrent UTIs
- Perineal ultrasound:
  4 Open bladder neck during filling phase
  5 Unstable (hypermobile) bladder neck during straining
- Urodynamic:
  6 Tower-shaped voiding curve during uroflowmetry
  7 Lag time <2 s.

## Conclusion

Urinary incontinence has a substantial negative impact on the quality of life and daily activities of affected individuals. Implementation of the proper diagnostic pathways followed by the adequate treatment of LUTD in childhood can possibly limit negative consequences in the long term. Establishing of the reliable criteria for the bladder neck insufficiency should be the goal of the future trials.

## Author Contributions

The author confirms being the sole contributor of this work and approved it for publication.

## Conflict of Interest Statement

The author declares that the research was conducted in the absence of any commercial or financial relationships that could be construed as a potential conflict of interest.
